# 2-Aminobutyric acid modulates glutathione homeostasis in the myocardium

**DOI:** 10.1038/srep36749

**Published:** 2016-11-09

**Authors:** Yasuhiro Irino, Ryuji Toh, Manabu Nagao, Takeshige Mori, Tomoyuki Honjo, Masakazu Shinohara, Shigeyasu Tsuda, Hideto Nakajima, Seimi Satomi-Kobayashi, Toshiro Shinke, Hidekazu Tanaka, Tatsuro Ishida, Okiko Miyata, Ken-ichi Hirata

**Affiliations:** 1 Division of Evidence-Based Laboratory Medicine, Kobe University Graduate School of Medicine, 7-5-1, Kusunokicho, Chuo-ku, Kobe, 650-0017, Japan; 2 The Integrated Center for Mass Spectrometry, Laboratory Medicine, Kobe University Graduate School of Medicine, 7-5-1, Kusunokicho, Chuo-ku, Kobe, 650-0017, Japan; 3 Division of Cardiovascular Medicine, Kobe University Graduate School of Medicine, 7-5-1, Kusunokicho, Chuo-ku, Kobe, 650-0017, Japan; 4 Division of Epidemiology, Kobe University Graduate School of Medicine, 7-5-1, Kusunokicho, Chuo-ku, Kobe, 650-0017, Japan; 5 Medicinal Chemistry Laboratory, Kobe Pharmaceutical University, 4-19-1, Motoyamakita, Higashinada, Kobe, 658-8558, Japan

## Abstract

A previous report showed that the consumption of glutathione through oxidative stress activates the glutathione synthetic pathway, which is accompanied by production of ophthalmic acid from 2-aminobutyric acid (2-AB). We conducted a comprehensive quantification of serum metabolites using gas chromatography-mass spectrometry in patients with atrial septal defect to find clues for understanding myocardial metabolic regulation, and demonstrated that circulating 2-AB levels reflect hemodynamic changes. However, the metabolism and pathophysiological role of 2-AB remains unclear. We revealed that 2-AB is generated by an amino group transfer reaction to 2-oxobutyric acid, a byproduct of cysteine biosynthesis from cystathionine. Because cysteine is a rate-limiting substrate for glutathione synthesis, we hypothesized that 2-AB reflects glutathione compensation against oxidative stress. A murine cardiomyopathy model induced by doxorubicin supported our hypothesis, i.e., increased reactive oxygen species are accompanied by 2-AB accumulation and compensatory maintenance of myocardial glutathione levels. Intriguingly, we also found that 2-AB increases intracellular glutathione levels by activating AMPK and exerts protective effects against oxidative stress. Finally, we demonstrated that oral administration of 2-AB efficiently raises both circulating and myocardial glutathione levels and protects against doxorubicin-induced cardiomyopathy in mice. This is the first study to demonstrate that 2-AB modulates glutathione homeostasis in the myocardium.

Heart failure is becoming a worldwide public health problem and still has no known cure. In addition, the escalating medical care costs for heart failure impose a significant economic burden on our society^1^,^2^. The development of novel strategies to detect and treat patients at an early stage of heart failure before irreversible damage has occurred is an urgent task. We comprehensively quantified water-soluble metabolites in the blood of atrial septal defect (ASD) patients using gas chromatography-mass spectrometry (GC-MS) to find clues for understanding myocardial metabolic regulation. Metabolomics provides a comprehensive analysis of the characteristics and the interaction of low-molecular weight metabolites under specific conditions^3^,^4^,^5^. Conversely, circulating metabolites can be influenced by various factors, including other conditions of interest, medical agents, and diet. To alleviate these limitations, we focused on serum metabolic profiling in patients with ASD. ASD is characterized by shunting across a defect in the interatrial septum, and a comparison before and after ASD closure in the same person allows to evaluate the impact of hemodynamic changes on circulating metabolic profiling. Furthermore, since patients in the absence of significant volume overload or arrhythmia generally do not require specific medical therapy, metabolic profiling in patients with ASD also has an advantage for reducing the influence of medications. In the present study, we found that circulating 2-aminobutyric acid (2-AB) levels alter depending on hemodynamic status in patients with ASD.

Reduced glutathione (GSH), which is a ubiquitous tripeptide thiol, plays an essential role in the maintenance of the intracellular redox state^6^. The dysregulation of GSH homeostasis is implicated in the pathophysiology of cardiac remodeling and dysfunction^7^,^8^,^9^. GSH is synthesized through consecutive reactions with γ-glutamylcysteine synthetase and glutathione synthetase^10^. Oxidative stress activates the GSH biosynthetic pathway to compensate for increased GSH consumption^6^,^11^. Previously, Soga *et al.* reported that the activation of GSH biosynthetic pathway simultaneously initiates the production of ophthalmic acid, a GSH analog, from 2-aminobutyric acid (2-AB) and that ophthalmic acid is a potential biomarker for hepatic GSH depletion following oxidative stress^12^. In contrast, the regulatory mechanism of 2-AB metabolism under oxidative stress remains unidentified. In this study, we sought to investigate the pathophysiological role of 2-AB focusing on GSH homeostasis.

## Results and Discussion

### Circulating 2-AB levels alter depending on hemodynamic status

**Figure 1:**
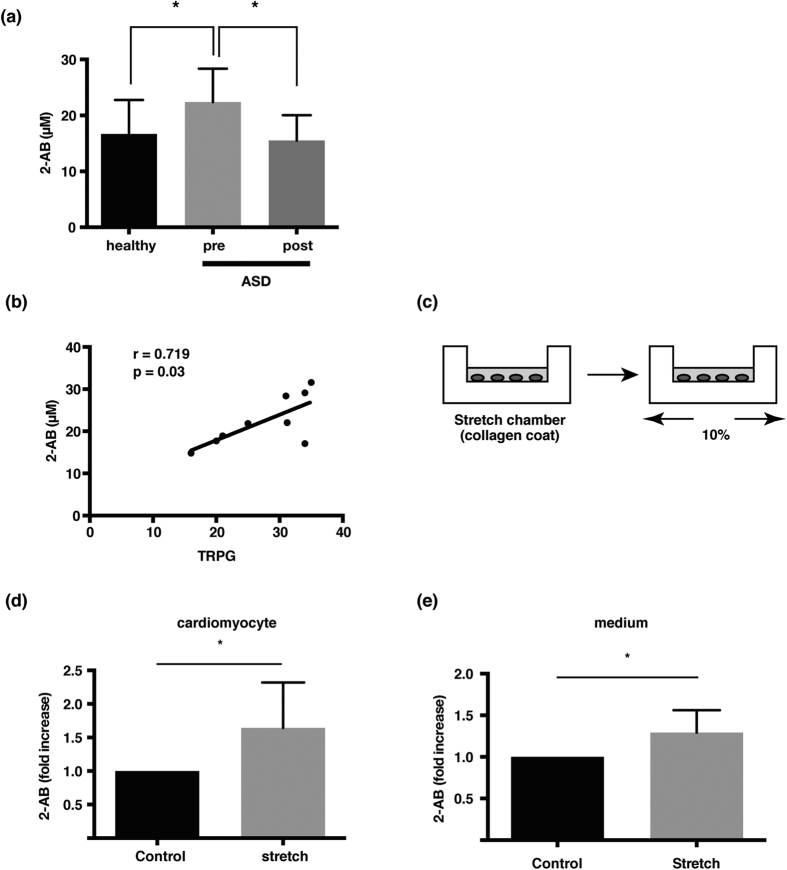
2-AB reflects excessive load conditions in hearts. (**a**) 2-AB levels in serum in healthy volunteers (n = 11) and ASD patients before (pre) and after (post) transcatheter closure (n = 8). (**b**) Correlation between 2-AB concentration and TRPG score. c, Illustration of stretch assay. H9c2 cells were stretched by 10% for 1 h. d, e, Mechanical stress induces 2-AB production by cells (**c**) and can be detected in the culture medium (**d**). (**e**) H_2_O_2_ increased intracellular levels of GSH in the cells. 2-AB, 2-aminobutyric acid; ASD, atrial septal defect; TRPG, tricuspid regurgitation peak gradient. *P < 0.05. P-values were determined by unpaired two-tailed Student’s *t*-tests.

Metabolome analysis in patients with ASD revealed that circulating 2-AB was significantly decreased 1 month after transcatheter closure of ASD among 85 metabolites (Supplementary Table 1). In addition, the mean level of 2-AB in ASD patients was increased compared with that in healthy volunteers. One month after closure of ASD, serum 2-AB levels in patients decreased to almost the same levels as in healthy volunteers (Fig. 1a). Subsequently, we examined the relationship between the levels of 2-AB and clinical data (Supplementary Table 2). Specifically, 2-AB concentrations were significantly correlated with tricuspid regurgitation peak gradient (TRPG) assessed by echocardiography, which is equal to a peak systolic pressure gradient between the right ventricle and the right atrium (Fig. 1b).

Because TRPG elevation reflects right-sided heart overload as a sequela of left-to-right shunting across the ASD, we examined whether mechanical stress to cardiomyocytes induces intracellular 2-AB accumulation. Differentiated H9c2 cardiomyocytes were plated on a collagen-coated chamber, and the cells were stretched by 10% for 1 h (Fig. 1c). This mechanical stress increased the 2-AB levels in H9c2 cells and the culture medium (Fig. 1d,e).

### 2-AB is an amino transfer enzyme-mediated byproduct in the cysteine biosynthesis pathway

**Figure 2:**
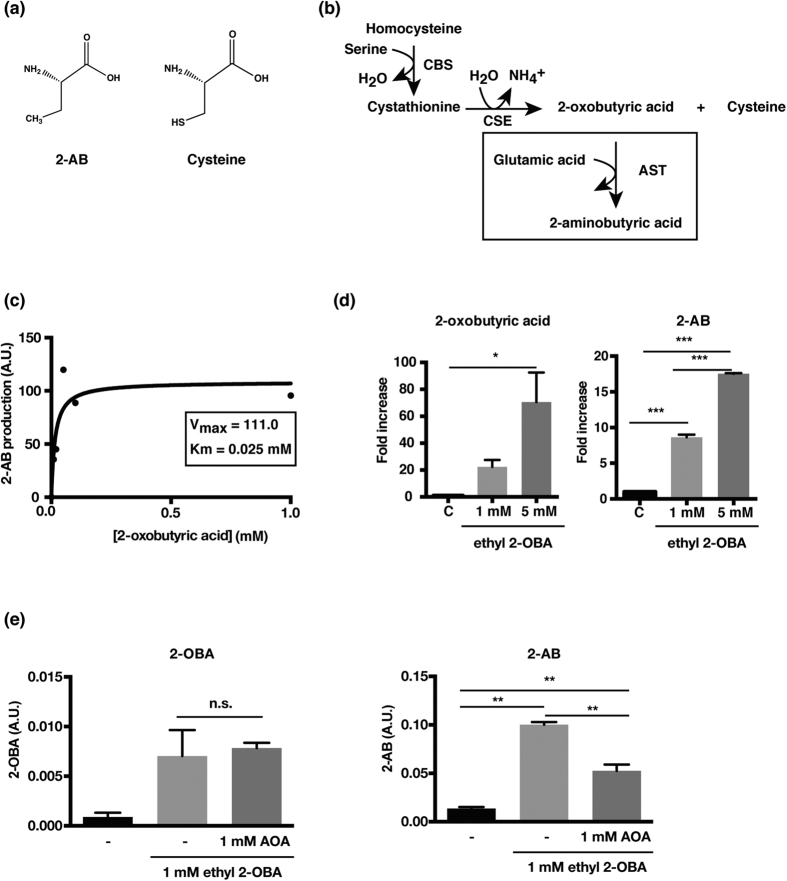
2-AB is synthesized via the cysteine metabolism pathway. (**a**) Structural formula of 2-AB and cysteine. (**b**) Schematic of cysteine metabolism pathway. (**c**) 2-AB production from 2-OBA. 2-OBA was incubated with glutamic acid and AST for 30 minutes. The reaction was stopped by adding HCl. GC-MS was used to measure 2-AB yield. (**d**) Intracellular 2-AB levels in H9c2 cells treated with ethyl 2-OBA. Cells were incubated with 1 mM or 5 mM ethyl-OBA for 1 h. After the incubation, the levels of 2-OBA and 2-AB were analyzed with GC-MS. Data are presented as the mean ± standard deviation (s.d.) of two independent experiments. *P < 0.05, ***P < 0.001. P-values were determined by ANOVA with Tukey’s multiple comparisons post-test. (**e**) Decreased 2-AB production in H9c2 cells treated with AOA, an inhibitor of AST. Cells were preincubated with 1 mM AOA for 1 h and incubated with 1 mM ethyl 2-OBA. The levels of 2-AB and 2-OBA were determined with GC-MS. Data are presented as the mean ± standard deviation (s.d.) of two independent experiments. **P < 0.01. n.s., not significant. P-values were determined by ANOVA with Tukey’s multiple comparisons post-test. A.U., arbitrary units; CBS, cystathionine beta synthase; CSE, cystathionine gamma lyase; 2-OBA, 2-oxobutyric acid; AOA, aminooxyacetic acid; AST, aspartate aminotransferase.

Next, we sought to identify the 2-AB synthesis pathway. Given that 2-AB has a similar structural formula to cysteine (Fig. 2a), we hypothesized that 2-AB is produced using the cysteine biosynthesis pathway. When cysteine is biosynthesized from cystathionine, 2-oxobutyric acid (2-OBA) is generated simultaneously (Fig. 2b). Considering the structure of 2-AB, we hypothesized that 2-AB is generated by an amino group transfer reaction to 2-OBA by amino transfer enzymes, such as aspartate aminotransferase (AST). To test this hypothesis, 2-OBA was incubated with glutamic acid and AST. We found that 2-AB was produced in accordance with increasing 2-OBA concentration (Fig. 2c). We then investigated whether 2-AB was generated from 2-OBA in cells. Because 2-OBA was not incorporated into the cells, we prepared ethyl 2-OBA to promote the incorporation into cells. We confirmed that 2-AB was biosynthesized from the incorporated 2-OBA (Fig. 2d). To examine the involvement of amino transfer enzymes for 2-AB biosynthesis, we used an inhibitor of AST, aminooxyacetic acid (AOA). Although AOA treatment had no effect on the incorporation of 2-OBA, 2-AB production was significantly suppressed (Fig. 2e). These results suggest that 2-AB is an AST-mediated byproduct in the cysteine biosynthesis pathway.

### GSH compensation against oxidative stress accompanies elevation of 2-AB

**Figure 3:**
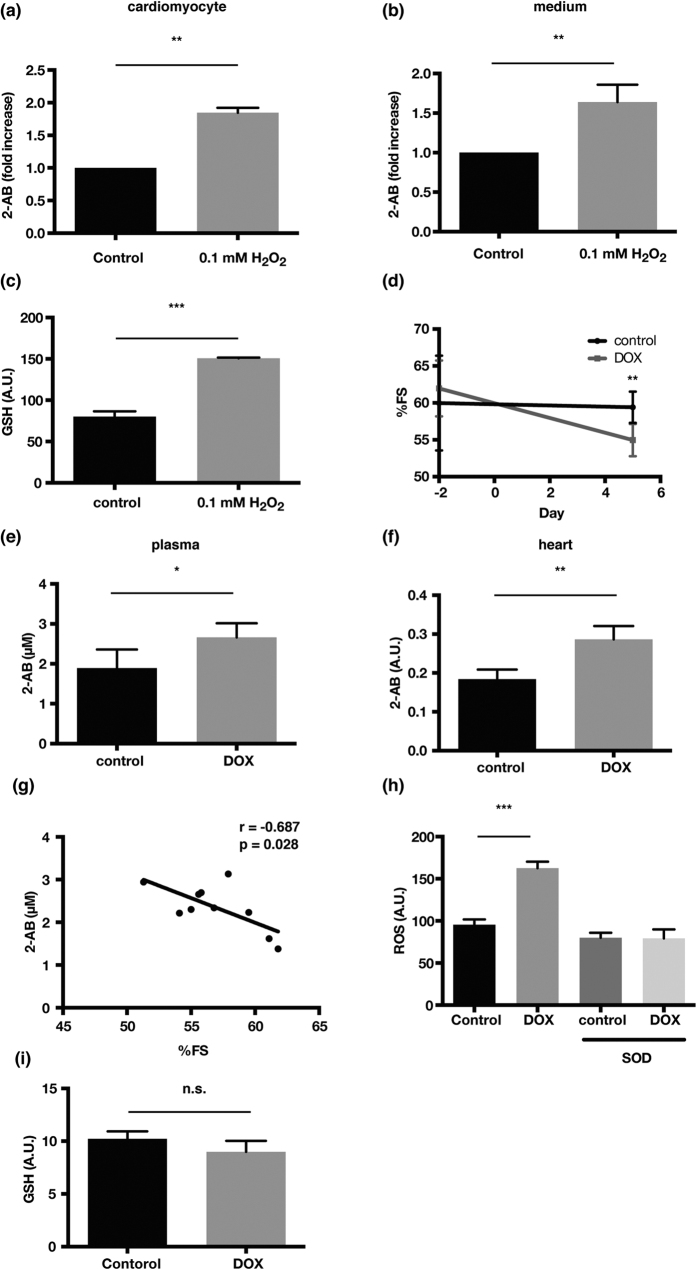
Oxidative stress leads to the accumulation of 2-AB in hearts, reflecting GSH homeostasis. (**a–c**) H_2_O_2_ increased intracellular levels of 2-AB (**a**) and GSH (**c**) in the cells as well as 2-AB levels in the medium (**b**). Cells were treated with 0.1 mM H_2_O_2_ for 24 h and the levels of 2-AB and GSH were measured. Bars represent the mean ± s.d. (n = 3). (**d**) %FS as assessed by echocardiography at 5 days is presented. e, f, Increased 2-AB levels in plasma (e) and hearts (**f**) in DOX-injected mice. Bars indicate the mean ± s.d. (n = 4, control; n = 6, DOX-injected mice in (**e**); n = 3, control; n = 7, DOX-injected mice in (**f**)). (**g**) Correlation between 2-AB concentration in plasma and %FS. (**h**) ROS production in control and DOX-injected heart homogenates detected by lucigenin-enhanced chemiluminescence in the presence or absence of SOD, a ROS scavenger. (**i**) Glutathione levels in heart tissues of DOX-injected mice (n = 4, control; n = 5 DOX-injected mice). %FS, percentage of fractional shortening; DOX, doxorubicin; ROS, reactive oxygen species; SOD, superoxide dismutase. *P < 0.05, **P < 0.01, ***P < 0.0001. n.s., not significant. P-values were determined by unpaired two-tailed Student’s *t*-tests.

Because cysteine is a rate-limiting substrate for *de novo* GSH synthesis^13^, we hypothesized that 2-AB reflects a compensatory maintenance of GSH under oxidative stress conditions. We found that oxidative stress caused by H_2_O_2_ administration increased the 2-AB levels in both cardiomyocytes and culture medium (Fig. 3a,b), depending on AST activity (Supplementary Figure 1). Oxidative stress also increased the total GSH levels, which suggests that GSH production is increased to counteract oxidative stress (Fig. 3c). Mechanical stress-induced 2-AB accumulation may also be mediated by oxidative stress, because stretch stress has been reported to increase ROS levels in cardiomyocytes^14^. We also assessed whether 2-AB reflects the myocardial redox state in murine models of cardiomyopathy induced by doxorubicin (DOX), which causes cardiac damage via oxidative stress. Echocardiographic analysis of cardiac function revealed that the administration of DOX decreased the percentage of fractional shortening (%FS) (Fig. 3d). The levels of 2-AB in both plasma and hearts were also increased after the administration of DOX (Fig. 3e,f). In addition, the 2-AB levels in plasma were associated with %FS (Fig. 3g). Reactive oxygen species (ROS) abundance was increased in DOX-treated hearts, consistent with previous reports (Fig. 3h). However, the total GSH levels were retained (Fig. 3i), which suggests that increased 2-AB reflected a compensation in GSH levels for consumption by ROS. Previous reports suggested that the detection of GSH deficiency in blood could be a novel strategy for the early diagnosis of heart failure^15^. However, because the extracellular GSH levels are 100- to 1000-fold lower than the intracellular levels^16^, we could not determine the circulating GSH levels using a popular enzymatic recycling assay. Moreover, as shown in the present study, the GSH levels do not necessarily decrease in parallel with ROS production because of its compensatory mechanism. On the other hand, the present findings suggest that the monitoring of circulating 2-AB levels has a potential to provide a better understanding of GSH homeostasis.

### 2-AB increases intracellular GSH levels and exerts protective effects against oxidative stress

**Figure 4:**
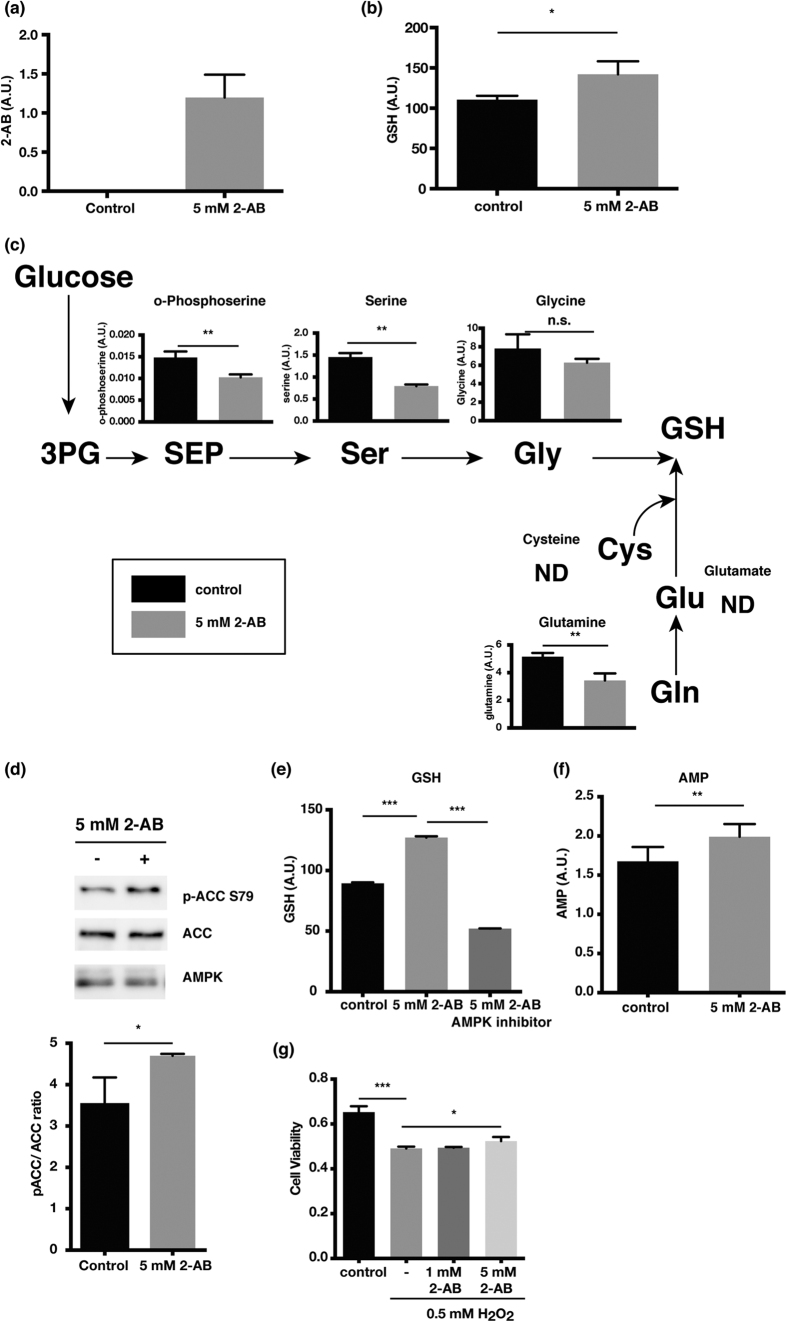
2-AB increases intracellular GSH levels by altering metabolism and AMPK activation as well as exerting cardioprotective effects against oxidative stress. (**a,b**) Intracellular 2-AB (**a**) and GSH (**b**) levels in H9c2 cells after incubation with 5 mM 2-AB for 30 min. (**c**) Intracellular metabolite levels in the partial metabolism of GSH. (**d**) Western blot of H9c2 cells incubated with 5 mM 2-AB. Phosphorylated ACC and ACC protein levels were quantified using Image Studio^TM^ software. Bars indicate the mean ± s.d. (n = 3). *P < 0.05. P-values were determined by unpaired two-tailed Student’s t-tests. (**e**) Effect of AMPK inhibitor on 2-AB induced GSH production. Cells were pretreated with 5 μM AMPK inhibitor for 4 h and incubated with 5 mM 2-AB for 30 min. Bars indicate the mean ± s.d. (n = 3). ***P < 0.0001. P-values were determined by ANOVA with Tukey’s multiple comparisons post-test. (**f**) Intracellular levels of AMP in 2-AB stimulated cells. The cells were incubated with 5 mM 2-AB for 30 min. The abundance of AMP was determined using a quantification kit. Bars indicate the mean ± s.d. (n = 7). **P < 0.01. P-values were determined by unpaired two-tailed Student’s t-tests. (**g**) Effect of 2-AB treatment on H9c2 cell viability. 3PG, glycerate-3-phosphate; SEP, o-phosphoserine; Ser, serine; Gly, glycine; GSH, glutathione; Cys, cysteine; Glu, glutamic acid; Gln, glutamine; ND, not detected; pACC, phosphorylated acetyl-CoA carboxylase; ACC, acetyl-CoA carboxylase. Bars indicate the mean ± s.d. (n = 3). *P < 0.05, **P < 0.01, ***P < 0.0001. n.s., not significant. P-values were determined by unpaired two-tailed Student’s t-tests.

Next, we investigated the physiological roles of 2-AB. When 2-AB was treated with H9c2 cells, 2-AB was easily incorporated into cells, the most probable mechanism being via amino acid transporters (Fig. 4a). Surprisingly, intracellular GSH levels were increased after incubation with 2-AB (Fig. 4b). To examine how 2-AB influences GSH levels in cells, we metabolically profiled 2-AB treated cells. Metabolic profiles of cardiomyocytes stimulated by 2-AB imply the activation of the serine biosynthesis pathway (Fig. 4c). A decrease in serine indicates the satisfaction of glycine requirements, which is mainly biosynthesized from serine and components of GSH. Moreover, serine is required for the reaction that produces cystathionine from homocysteine. Glutamine, a precursor of glutamic acid, was also decreased. Although we cannot conclude without metabolic flux analysis, these results might reflect that 2-AB alters metabolism to meet the demands of the intermediates for GSH synthesis.

Recent studies have revealed that AMPK has a key function in nicotinamide adenine dinucleotide phosphate (NADPH) maintenance^17^. The oxidized form of GSH (GSSG) is reduced with NADPH. Thus, we hypothesized that 2-AB regulates AMPK activity. In support of this notion, AMPK was activated in H9c2 cells treated with 2-AB (Fig. 4d). As further confirmation of the involvement AMPK on GSH production induced by 2-AB, we inhibited AMPK using an AMPK inhibitor and assessed GSH productivity of 2-AB. Remarkably, the treatment of AMPK inhibitor decreased GSH production (Fig. 4e). Given that AMPK allosterically activated by AMP^18^, we measured the levels of AMP in 2-AB treated cells. The treatment of 2-AB increased the intracellular levels of AMP (Fig. 4f). Together, these data support the idea that 2-AB regulates AMPK activity to increase the GSH levels.

To test whether 2-AB mediated elevation of GSH possesses cell protective effects, we examined the roles of 2-AB in cell viability. Pretreatment with 2-AB suppressed the cell death observed when H9c2 cardiomyocytes were incubated with H_2_O_2_ (Fig. 4g).

**Figure 5:**
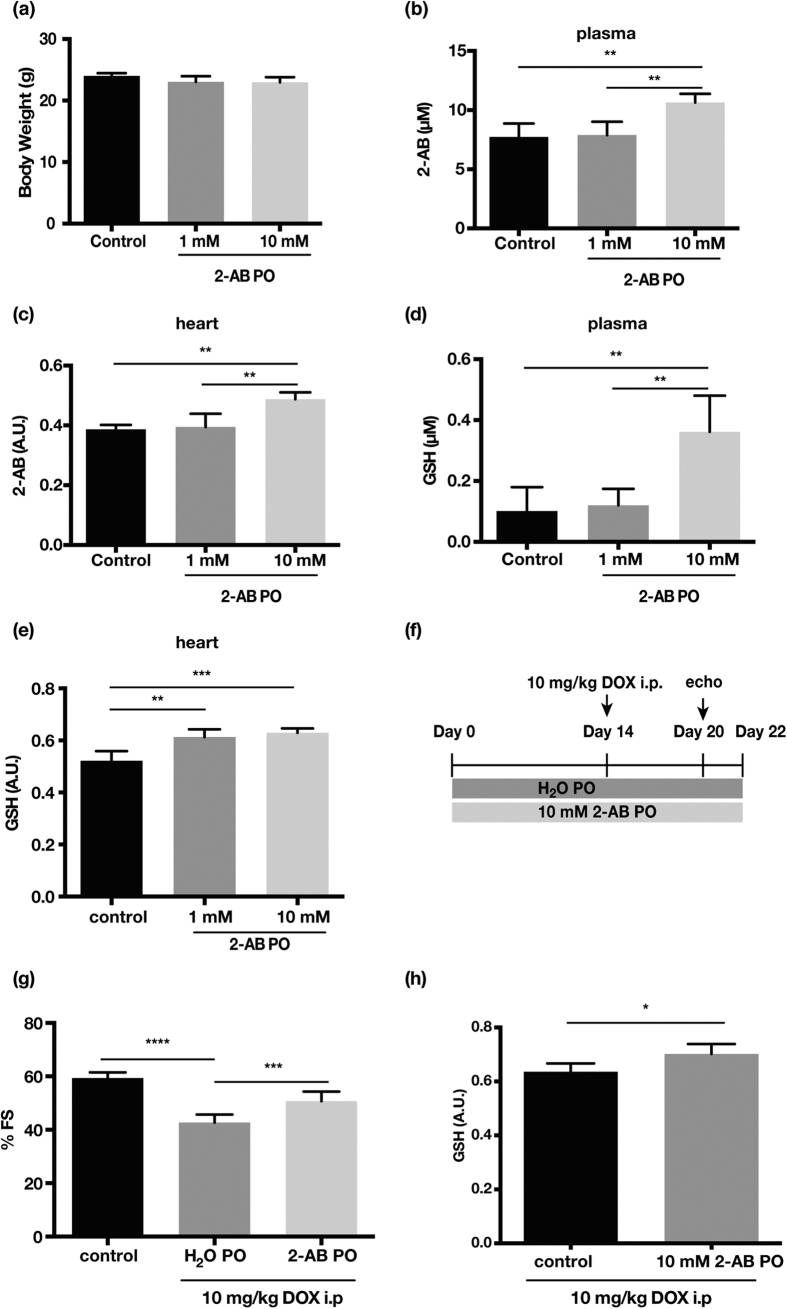
Oral administration of 2-AB leads to elevated 2-AB as well as GSH in plasma and hearts, and exerts a cardioprotective effect. (**a**) Body weight of 2-AB treated mice after 1 week oral administration of 2-AB. Bars indicate the mean ± s.d. (n = 5, control and 1 mM 2-AB treated mice; n = 4, 10 mM 2-AB treated mice). (**b,c**) The levels of 2-AB in plasma (**b**) (n = 4, control, n = 5, 1 mM 2-AB treated mice, n = 4, 10 mM 2-AB treated mice) and hearts (**c**) (n = 5, control and 1 mM 2-AB treated mice; n = 4, 10 mM 2-AB treated mice) with GC-MS analysis. (**d,e**) The levels of GSH in plasma (**d**) and hearts (**e**) (n = 4, control, n = 5, 1 mM 2-AB treated mice and 10 mM 2-AB treated mice). (**f**) Chronic doxorubicin (DOX)-induced cardiomyopathy was induced with 10 mg/kg intraperitoneal injection (i.p.) of DOX as shown. Before the DOX injection, mice were administrated orally for 14 days with the vehicle solution or 10 mM 2-AB solution. (**g**) Fractional shortening (FS) as assessed by echocardiography 6 days after the initial DOX injection. (**h**) The levels of GSH in hearts in DOX-injected mice (n = 4, each group). PO, per ou. Bars indicate the mean ± s.d. *P < 0.05, **P < 0.01, ***P < 0.0001.

To confirm 2-AB mediated GSH production *in vivo*, 2-AB was administered to mice in drinking water for 1 week. Oral administration of 2-AB had no effect on body weight (Fig. 5a). The amounts of 2-AB were increased in both plasma and hearts in a dose-dependent manner (Fig. 5b,c). Coincident with 2-AB increase, GSH levels in plasma and hearts increased (Fig. 5d,e). Finally, we also sought to demonstrate the cardioprotective effect of 2-AB *in vivo* (Fig. 5f). Pretreatment with 2-AB protected against DOX-induced cardiomyopathy in mice concomitantly with elevation of myocardial GSH levels (Fig. 5g,h).

Recently, the elevation of 2-AB levels in heart tissue has also been observed in global metabolomics analysis of a hamster model for dilated cardiomyopathy^19^, supporting the notion that 2-AB could facilitate early detection of heart failure. Exogenous GSH preserves mitochondrial energetic/redox balance and exerts cardioprotective effects^20^,^21^,^22^,^23^. However, oral administration of GSH does not efficiently increase circulating levels of GSH^24^,^25^,^26^. Whereas 2-OBA requires esterification for cellular uptake, 2-AB is incorporated directly into cells, followed by an increase in intracellular GSH levels. Furthermore, oral intake of 2-AB raised both circulating and myocardial GSH levels and provided a cardioprotective effect. Taken together, the current findings suggest the potential of 2-AB modulation as a novel therapeutic strategy for targeting dysregulation in cellular GSH homeostasis.

## Methods

### Human study

This study was approved by the Institutional Review Board of Kobe University Graduate School of Medicine and conducted according to the principles expressed in the Declaration of Helsinki. All patients and healthy volunteers provided their written informed consent.

### Quantification of 2-AB levels in serum

Metabolites were extracted from serum as previously described^27^. Analysis of 2-AB by GC-MS was performed on a GCMS-QP2010 Ultra (Shimadzu). The data were acquired with selected ion monitoring and calibrated using the peak height of 2-isopropylmalic acid (internal standard).

### Measurement of metabolites in cells

Cell metabolites were extracted with cold methanol. After centrifugation to remove cell debris, the metabolites were freeze-dried overnight. Lyophilized metabolites were derivatized and analyzed with GC-MS as previously described^28^.

Cell debris was lysed in 20 mM HEPES (pH 7.4), 150 mM NaCl, 1% NP-40, and 1% sodium dodecyl sulfate (SDS). The protein concentration of the lysate was measured with a commercially available BCA Protein Assay kit (Pierce). Metabolite measurements were normalized to protein content.

### Cell culture

H9c2 cells were purchased from the European Collection of Cell Culture (ECACC) and maintained in Dulbecco’s modified Eagle’s medium (DMEM) medium (WAKO) supplemented with 10% fetal bovine serum (FBS). The medium was changed to DMEM supplemented 1% FBS for differentiation.

### Mechanical stress assay

H9c2 cells were grown in DMEM with 10% FBS in collagen coated stretch chambers (STREX, Inc). The cells were differentiated with DMEM 1% FBS and stretched by 10% for 1 h.

### Animal experiments

The care and use of the animals were followed the animal welfare guidelines, and all the experimental protocols were approved the committee of Kobe University. Male C57/BL6J mice (10 weeks old) were purchased from CLEA Japan, Inc. and maintained and treated under specific pathogen-free conditions. 2-AB was orally administrated in drinking water for 1 week. Animals were euthanized, and their hearts and plasma were extracted.

For the doxorubicin (DOX)-induced cardiomyopathy models, 10 week old C57/BL6J mice were administrated a single injection (10 mg/kg intraperitoneally). A 10 mM 2-AB solution was administrated orally 14 days before DOX injection and 8 days after DOX injection.

Reactive oxygen species (ROS) levels were measured with lucigenin-enhanced chemiluminescence method as previously described^29^. Briefly, heart homogenates were incubated with 5 μM lucigenin, 100 μM NADPH in the presence or absence of superoxide dismutase (SOD), a ROS scavenger. Light emission was recorded with a 96-well microplate luminometer.

### 2-AB metabolism *in vitro* and *in vivo*

For *in vitro* synthesis of 2-AB, 2-oxobutyric acid was mixed with 50 mM Tris-HCl, 0.1 mM pyridoxal-5-phosphate, 0.1 mM glutamic acid, and 5 units of aspartate aminotransferase (WAKO). After incubation at 30 °C for 30 min, the reaction was stopped by adding 0.1 N HCl. GC-MS was used to measure 2-AB yield.

For *in vivo* synthesis of 2-AB, H9c2 cells were treated with 1 mM or 5 mM ethyl 2-OBA and incubated for 1 h. The production of 2-AB was measured using GC-MS.

### Cell viability assay

H9c2 cells were seeded in 96-well plates, and cells were differentiated in DMEM containing 1% FBS. The cells were treated with either water (control) or 2-AB at varying concentrations overnight. The cells were then stimulated with 0.5 mM H_2_O_2_ for 24 h, and the number of cells was determined with a Cell Counting Kit-8 (Dojindo) according to the manufacturer’s protocol.

### Measurement of glutathione

Glutathione levels in mouse plasma were determined using liquid chromatography–tandem mass spectrometry (AB SCIEX 6500 Qtrap) as described previously^30^. Total glutathione level in hearts and H9c2 cells was measured with a commercially available kit (Cayman Chemical) according to the manufacturer’s protocol. For AMPK inhibition experiments, cells were pretreated with 5 μM AMPK inhibitor for 4 h and incubated with 5 mM 2-AB for 30 min.

### Measurement of AMP

H9c2 cells were treated with 5 mM 2-AB for 30 min. The cells were washed with phosphate buffered saline and collected in 20 mM HEPES (pH 7.4), 150 mM NaCl and 1% NP40. The AMP levels in cell lysate were determined with a commercially available kit (Promega) according to the manufacturer’s instructions.

### Preparation of ethyl 2-oxobutyrate

According to a previously described procedure^31^, ethyl iodide (0.3 mL, 4 mmol) was added to a solution of sodium 2-oxobutyrate (248 mg, 2 mmol) in hexamethylphosphoramide (HMPA) (3 mL) at room temperature. After being stirred for 1 h, the reaction mixture was diluted with 5% HCl and extracted with Et_2_O. The combined organic phase was washed with 10% aqueous Na_2_S_2_O_3_ followed by saturated aqueous NaCl, and dried over MgSO_4_. The organic phase was concentrated under reduced pressure and the resulting residue was purified by flash column chromatography on silica gel [pentane:Et_2_O (10:3)] to produce ethyl 2-oxobutyrate (60 mg, 23%). The spectral data were identical with those reported in the literature^32^,^33^.

### Statistics

An unpaired two-tailed Student’s *t*-test or one-way analysis of variance (ANOVA) with Tukey’s post-hoc test was used to make comparisons as indicated. The relationship between the 2-AB levels and clinical data was analyzed using Pearson’s correlation. All statistical analyses were performed using GraphPad Prism software.

## Additional Information

**How to cite this article**: Irino, Y. *et al.* 2-Aminobutyric acid modulates glutathione homeostasis in the myocardium. *Sci. Rep.* **6**, 36749; doi: 10.1038/srep36749 (2016).

**Publisher’s note:** Springer Nature remains neutral with regard to jurisdictional claims in published maps and institutional affiliations.

## Supplementary Material

Supplementary Information
