# Supplementary material for: 2-Aminobutyric acid modulates glutathione homeostasis in the myocardium
Source: Sci Rep. 2016 Nov 9;6:36749. doi: 10.1038/srep36749 (PMC5101505; doi:10.1038/srep36749)
Supplement: Supplementary Information [file srep36749-s1.pdf]

Supplementary Information

2-Aminobutyric acid modulates glutathione homeostasis in the myocardium

Yasuhiro Irino, Ryuji Toh, Manabu Nagao, Takeshige Mori, Tomoyuki Honjo, Masakazu Shinohara, Shigeyasu Tsuda, Hideto Nakajima, Seimi Satomi-Kobayashi, Toshiro Shinke, Hidekazu Tanaka, Tatsuro Ishida, Okiko Miyata, and Ken-ichi Hirata

Supplementary table1.

A list of metabolites detected in the serum of ASD patients before and after the treatment

	Fold induction	P value
1,5-Anhydro-D-glucitol	0.94	0.110
1,6-Anhydroglucose	2.70	0.332
1-Hexadecanol	1.02	0.759
2,3-Bisphospho-glycerate	1.13	0.804
2-Aminobutyric acid	0.65	0.007
2-Aminoethanol	0.84	0.014
2-Aminoisobutyrate	1.08	0.231
3-Hydroxy-Butyrate	0.28	0.021
3-Hydroxyisovaleric acid	0.72	0.519
Acetylsalicylic acid	0.13	0.029
Aconitate	1.18	0.546
Alanine	0.99	0.899
Arabinose	1.05	0.426
Arabitol	1.79	0.250
Ascorbic acid	0.90	0.676
a-Sorbopyranose (or Fructose)	1.00	0.968
Asparagine	0.73	0.094
Aspartic acid	0.96	0.895
b-Alanine	1.01	0.893
Citric acid + Isocitric acid	0.88	0.178
Citrulline	0.93	0.719
Creatinine	0.89	0.408
Cysteine+Cystine	0.71	0.551
Fructose	1.10	0.549
Fumaric acid	1.12	0.511
Galactosamine	0.71	0.345
Galactose	1.02	0.937
Glucarate	1.18	0.304

Glucose	1.00	0.970
Glucuronate	0.78	0.040
Glutamic acid	1.07	0.825
Glutamine	0.91	0.581
Glyceraldehyde	0.94	0.326
Glyceric acid	1.16	0.549
Glycerol	0.49	0.042
Glycine	0.87	0.186
Glycolic acid	0.97	0.816
HydroxyButyrate	0.55	0.005
Hypoxanthine	0.81	0.640
Inositol	1.08	0.028
Isoleucine	0.86	0.152
Ketoisoleucine	0.96	0.617
Kynurenate	1.04	0.526
Lactic acid	1.04	0.702
Lauric acid	0.68	0.095
Lysine	0.83	0.042
Lyxose (or Xlylose)	1.02	0.941
Lyxose	1.03	0.881
Malic acid	0.93	0.568
Mannitol	0.55	0.162
Mannose	0.91	0.005
meso-erythritol	1.19	0.069
Methionine	0.88	0.418
N-a-Acetyl-L-Lysine	1.22	0.587
n-Butylamine	0.95	0.435
n-Caprylic acid	0.84	0.437
Nonanoic acid	0.78	0.083
O-Phosphoethanolamine	0.81	0.110
Ornithine	0.92	0.446
Oxalate	0.99	0.888
Palmitoleate	0.30	0.009

Phenylalanine	0.92	0.351
Phosphate	0.95	0.531
Proline	1.19	0.089
Psicose	4.99	0.073
Pyrogallol	0.34	0.444
Pyroglutamic acid	0.96	0.621
Pyruvate+Oxalacetic acid	0.84	0.622
Rhamnose	1.76	0.155
Ribitol	1.74	0.019
Ribose	0.52	0.302
Ribulose	0.59	0.009
Sarcosine	1.18	0.196
S-Benzyl-L-Cysteine	1.63	0.259
Serine	0.93	0.622
Succinic acid	0.91	0.697
Threitol	0.86	0.302
Threonine	0.78	0.030
trans-4-Hydroxy-L-proline	1.28	0.106
Tryptophan	1.14	0.473
Tyrosine	0.85	0.210
Uric acid	0.90	0.414
Valine	0.93	0.253
Xylitol	1.03	0.911
Xylose	1.18	0.569

Values are represented as the fold-induction in the peak intensity (post-treatment/ pre-treatment) (n=8). P values were calculated using the paired t-test.

Supplementary Table 2.

Clinical characteristics of patients with atrial spetal defect (ASD)

Sample No.	Sex	Age	Qp/Qs pre	TRPG pre	BNP pre	BNP post
1	M	78	1.9	34	67.8	76.0
2	M	58	3.5	35	182	315
3	F	45	1.8	31	21.6	43.5
4	F	17	1.2	16	20.7	19.8
5	M	49	2.2	34	35.4	51.6
6	M	66	3.1	25	16.2	31.7
7	F	71	1.2	31	86.4	73.6
8	F	17	2.4	21	29.4	24.5

M, male; F, female; Qp/Qs, pulmonary-systemic shunt ratio; TRPG, tricuspid regurgitation peak gradient; BNP, brain natriuretic peptide levels in serum; pre, before closure of ASD; post, one month after closure of ASD.

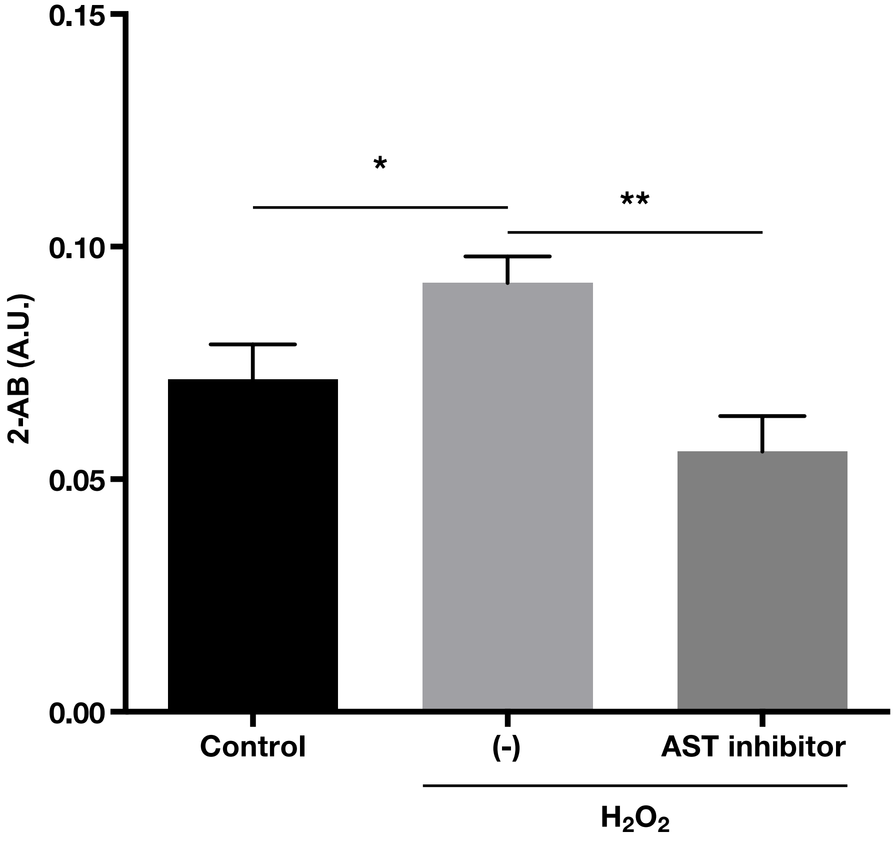

Supplementary Figure 1. The effect of AST inhibitor on 2-AB production in H_2O_2 treated cells. H9c2 cells were preincubated with 1 mM AST inhibitor for 1 h and incubated with 0.1 mM H_2O_2 for 6 h. The levels of 2-AB were measured with GC-MS analysis and normalized to protein content. Bars indicate the mean ± s.d. (n = 2, control; n=3, H_2O_2 treated cell; n = 4, H_2O_2 and AST inhibitor treated cell) *P < 0.05, **P < 0.01. P-values were determined by ANOVA with Tukey' s multiple comparisons post-test. A.U., arbitrary units.
